# Personality Traits Predict Weight Loss Following Endoscopic Sleeve Gastroplasty: A Cohort Study with External Validation

**DOI:** 10.1007/s11695-026-08504-9

**Published:** 2026-03-28

**Authors:** Benjamin I. Richter, Sameer Rao, Lucie Pham, Emmanuel Attah, Ana Hamann, Kyle Liang, Kaveh Hajifathalian, Reem Z. Sharaiha

**Affiliations:** 1 Department of Medicine, Rutgers, The State University of New Jersey, Newark, NJ United States; 2 Department of Medicine, NewYork–Presbyterian Hospital, New York, NY United States

**Keywords:** Endoscopic sleeve gastroplasty, Personality traits, Emotional stability, Big Five personality traits, Bariatric endoscopy

## Abstract

**Background:**

Endoscopic sleeve gastroplasty (ESG) is a safe and effective treatment for obesity, but weight loss varies widely among patients. Personality traits may influence behavioral adherence after bariatric interventions. We evaluated whether Big Five personality traits predict weight loss after ESG and validated findings across two demographically distinct cohorts.

**Methods:**

Two retrospective cohorts were analyzed. The primary cohort included self-pay patients who underwent ESG at a tertiary center in New York (2013–2019). The external cohort included predominantly uninsured or underinsured patients treated at a New Jersey academic/safety-net hospital (2021–2024). Patients completed the Ten-Item Personality Inventory (TIPI). Weight loss was measured as percent total body weight loss (%TBWL). Associations between personality traits and %TBWL were assessed using multilevel mixed-effects linear regression.

**Results:**

Primary cohort: Thirty-four patients (83% female, mean age 48±13; baseline BMI 36±4 kg/m²) completed TIPI with median follow-up of 24 months. Mean %TBWL was 15.7±6.2% (Table 1). Emotional stability was significantly associated with greater %TBWL, with each one-point increase corresponding to 1.9% higher TBWL (95% CI 0.2–3.5%; p=0.026). The association persisted after adjusting for baseline BMI and follow-up compliance (β=1.9; 95% CI 0.4–3.3%; p=0.017). No other traits were significant predictors (Table 2). External validation cohort: Twenty-three patients (74% female, mean age 47±14; baseline BMI 44±4 kg/m²) completed TIPI with median follow-up of 17 months. Mean %TBWL was 12.6±12.2% (Table 1). Emotional stability again predicted%TBWL, with each one-point increase associated with 3.4% greater TBWL (95% CI 0.1–6.5%; p=0.042). No other traits were significant predictors (Table 2).

**Conclusion:**

Across two demographically and socioeconomically distinct cohorts, emotional stability was the only personality trait consistently associated with weight loss after ESG. This trait—reflecting resilience to stress and effective emotional regulation—may support long-term adherence to post-ESG behavioral changes. The TIPI enables rapid, point-of-care personality assessment and can facilitate personalized, risk-stratified counseling. Patients with lower emotional stability may benefit from enhanced behavioral or psychological support. Further prospective studies are needed to clarify causal mechanisms and optimize pre-procedural risk stratification.

## Introduction

Obesity affects over one billion people worldwide, representing a growing public health crisis [1]. Endoscopic sleeve gastroplasty (ESG) is a minimally invasive, safe, and effective treatment option for obesity, but outcomes vary, prompting efforts to identify phenotypes that predict weight loss [2–4].

Personality traits may influence outcomes following bariatric interventions [5, 6]. The Five-Factor Model—encompassing emotional stability, agreeableness, extraversion, conscientiousness, and openness—is a personality framework that can be evaluated using a brief, validated survey at the point of care. Emotional stability reflects emotional regulation and stress tolerance; agreeableness reflects interpersonal warmth and cooperativeness; conscientiousness reflects self-discipline and goal-directed behavior; extraversion reflects sociability and positive affect; and openness reflects curiosity and receptivity to new experiences. The Ten-Item Personality Inventory (TIPI) assesses each trait using two items on a 1–7 Likert scale (1 = low, 7 = high trait expression). Higher scores indicate greater expression of each trait. In the context of obesity treatment, personality traits may plausibly impact behavioral adherence to dietary and lifestyle choices and coping with stress-related eating. We hypothesized that personality traits are associated with weight loss following ESG and sought to validate these findings across demographically distinct cohorts.

## Methods

### Study Population and Follow-up

#### Primary Cohort

**Fig. 1:**
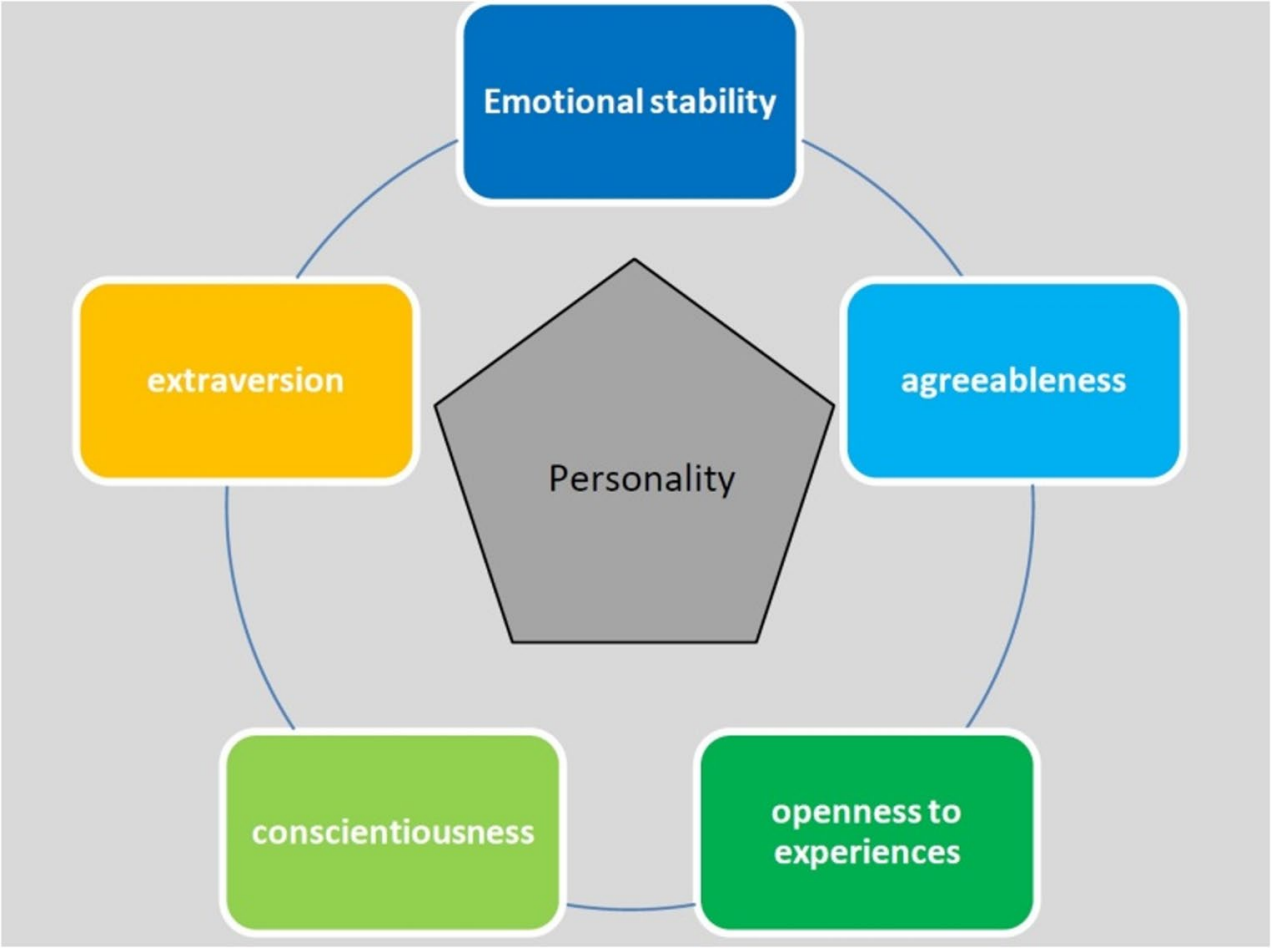
Big-Five dimensions of personality

This cohort included patients who underwent ESG from August 2013 to August 2019 at a tertiary academic medical center in New York City where most patients were self-pay. All procedures were performed in a single center by the same gastroenterologist (R.S.) under institutional review board approval (IRB Protocol 1510016654). A consecutive list of patients from our endobariatrics registry completed the Ten-Item Personality Inventory (TIPI), with inclusion based on availability and willingness to participate, to assess Big Five traits (Fig. 1).

Personality traits—enduring patterns of thought, emotion, and behavior—are described by the Big Five model: Openness, Conscientiousness, Extraversion, Agreeableness, and Emotional Stability. This framework is cross-culturally validated and incorporated into the DSM-5-TR [7]. The TIPI provides a brief, validated, patient-reported assessment of these traits with acceptable psychometric properties, offering structural validity while minimizing participant burden [8].

Inclusion criteria were BMI > 30 kg/m² and failure of prior noninvasive weight loss interventions. Patients with BMI > 40 kg/m² were eligible if they declined or were ineligible for bariatric surgery. Exclusion criteria included prior bariatric procedures, family history of gastric cancer, history of gastric neoplasia, significant psychiatric illness, coagulopathy, or comorbidities precluding deep sedation. Patients were evaluated by a multidisciplinary team and were not required to discontinue weight loss pharmacotherapy post-ESG.

Anthropometric and laboratory data were collected at baseline and at 1-, 3-, 6-, and 12-months post procedure, with annual follow-up encouraged. All patients received standardized dietary and lifestyle counseling.

#### External Validation Cohort

This cohort included patients who underwent ESG between September 2021 and December 2024 at the primary safety-net hospital in New Jersey serving predominantly uninsured and underinsured populations. All procedures were performed by a single gastroenterologist (K.H.) under IRB approval (Pro2024002500). All other methods mirrored the primary cohort.

### Procedure and Outcomes

ESG techniques and postoperative dietary protocols followed published standardized methods [3].

Weight loss was measured as percent total body weight loss (%TBWL = [(Initial Weight – Post-op Weight)/Initial Weight] × 100). Personality traits were assessed using the TIPI, with each trait scored on a 1–7 Likert scale, at the time of last available follow up. The primary outcome was %TBWL at the last available follow-up among patients with at least 12 months of follow-up. Use of AOMs was permitted according to a similar protocol in both institutions, so weight loss outcomes do not necessarily represent the effect of the ESG alone.

### Statistical Analysis

Descriptive statistics are reported as mean (standard deviation, SD) and median (interquartile range, IQR). Multilevel mixed-effects linear regression models were fit for each personality trait separately, with time included as a fixed effect and with patient-level random intercepts, to evaluate associations between personality traits and weight loss. All tests were two-tailed with α = 0.05.

## Results

### Primary Cohort

Thirty-four patients (83% female, mean age 48 ± 13 years) completed the TIPI. Mean Baseline BMI was 36 ± 4 kg/m². Median follow-up duration was 24 months (IQR 12–24). Mean %TBWL was 15.7 ± 6.2% (Table 1). The highest mean personality score was for conscientiousness (5.5 ± 1.3); lowest for agreeableness (4.6 ± 1.3).

**Table 1:** Baseline characteristics of study cohorts

Characteristic	Primary cohort (*n* = 34)	External validation cohort (*n* = 23)
Age, years (mean ± SD)	48 ± 13	47 ± 14
Female sex, n (%)	28 (83%)	17 (74%)
Baseline BMI, kg/m² (mean ± SD)	36 ± 4	44 ± 4
Follow-up duration, months (median [IQR])	24 [IQR: 12–24]	17 [IQR 14–22]
Mean personality trait scores (TIPI)		
Extraversion	4.9 ± 1.7	4.6 ± 1.4
Agreeableness	4.6 ± 1.3	6.0 ± 1.0
Conscientiousness	5.5 ± 1.3	5.6 ± 1.5
Emotional stability	5.1 ± 1.3	5.1 ± 1.6
Openness	4.7 ± 1.1	5.5 ± 1.7

*BMI* body mass index, *TIPI* Ten-Item Personality Inventory, *IQR* interquartile range, *SD* standard deviation

**Fig. 2:**
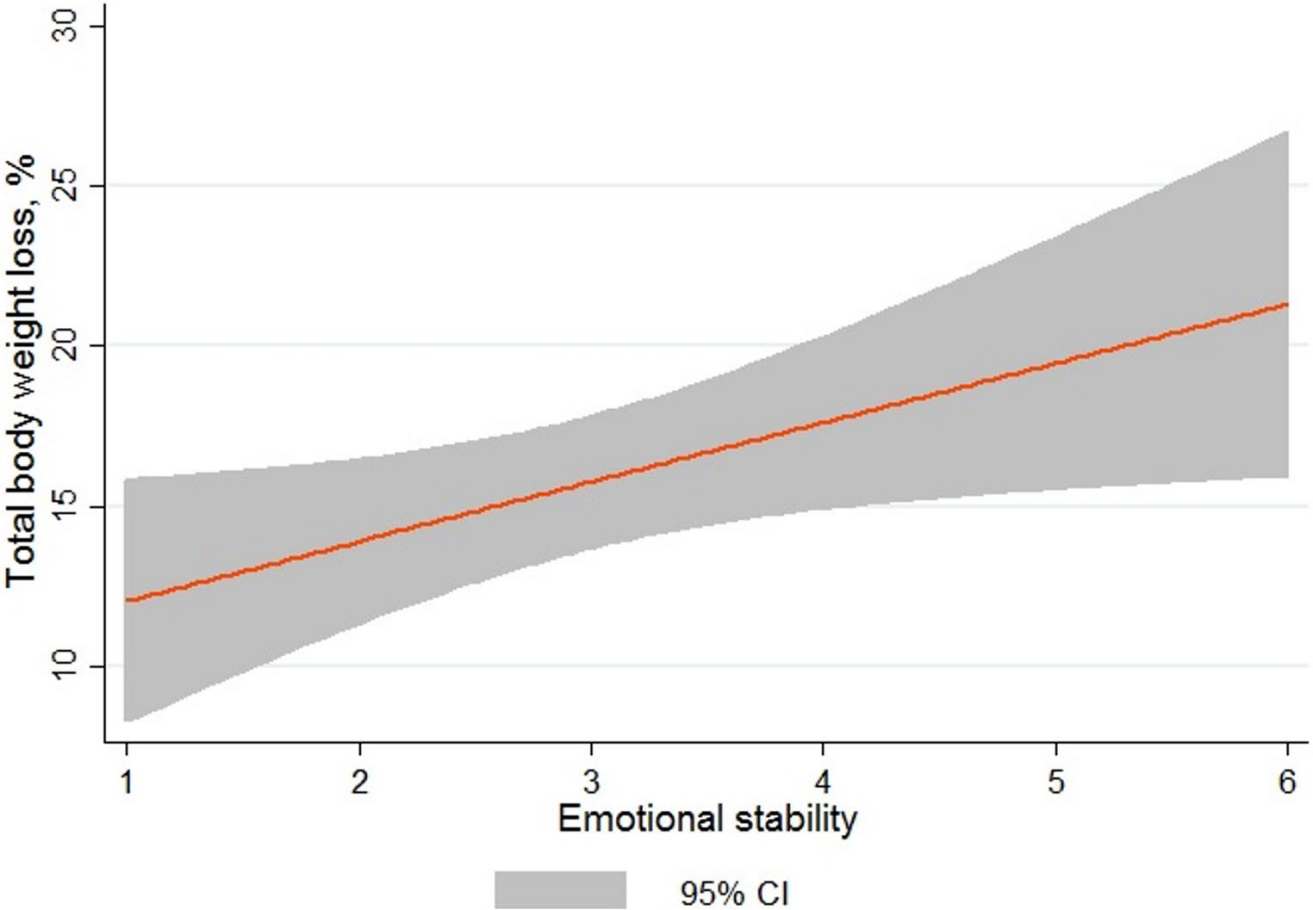
The association between emotional stability and total body weight loss after endoscopic sleeve gastroplasty (each one unit increase in emotional stability associated with 1.9% more TBWL; 95%CI 0.2 to 3.5%, *p*=0.026)

Emotional stability demonstrated a significant positive association with %TBWL, with each one-point increase in emotional stability score corresponding to 1.9% greater TBWL (95% CI 0.2–3.5%, *p* = 0.026) (Fig. 2). This association remained statistically significant after adjusting for baseline BMI and follow-up compliance (β = 1.9, 95% CI 0.4–3.3%, *p* = 0.017). No other domains were associated with weight loss (Table 2).

**Table 2:** Weight loss and personality trait outcomes

Outcome measure	Primary cohort (*n* = 34)	External validation cohort (*n* = 23)
% Total body weight loss (%TBWL, mean ± SD)	15.7 ± 6.2	12.6 ± 12.2
Association of Personality Trait (TIPI) with %TBWL (β, 95% CI, p-value)		
Extraversion	0.25 (−1.07- 1.56), *p* = 0.70	3.65 (–0.06 to 7.37), *p* = 0.054
Agreeableness	1.25 (−0.47–2.99), *p* = 0.15	1.67 (–3.83 to 7.16), *p* = 0.535
Consciensciousness	0.19 (−1.56–1.94), *p* = 0.83	2.12 (–1.38 to 5.63), *p* = 0.221
Emotional stability	**1.85 (0.23–3.47)**, *p* = 0.03	**3.35 (0.12 to 6.59)**, *p* = **0.043**
Openness	0.58 (−1.48–2.64), *p* = 0.57	–0.42 (–3.69 to 2.86), *p* = 0.793

*TIPI* Ten-Item Personality Inventory, *β* regression coefficient, *CI* confidence interval, *%TBWL* percent total body weight loss

Bold indicates statistical significance (*p* < 0.05)

Separate linear regression models were performed for each personality trait

### External Validation Cohort

Twenty-three patients (74% female; mean age 47 ± 14 years) completed the TIPI. Baseline BMI was 44 ± 4 kg/m², with median follow-up of 17 months (IQR 14–22). Mean %TBWL was 12.6 ± 12.2% (Table 1). The highest mean personality score was for agreeableness (6.0); lowest for extraversion (4.6).

Emotional stability was again significantly associated with %TBWL, with each one-point increase corresponding to 3.4% greater TBWL (95% CI 0.1–6.5%, *p* = 0.042). No other domains were associated with weight loss (Table 2).

## Discussion

Emotional stability was consistently the only personality domain predictive of post-ESG weight loss across two demographically and socioeconomically distinct cohorts, suggesting a robust and generalizable relationship. This trait—characterized by resilience to stress and effective emotional regulation—may enhance adherence to post-ESG behavioral changes, like sustained dietary modification, physical activity, and follow-up attendance. While results are mixed, several prior bariatric surgery studies have linked lower emotional stability to poorer postoperative outcomes, although heterogeneity across studies and limited sample sizes may obscure this relationship [5, 6, 9].

Personality traits are generally considered fixed over time and therefore our administration of the TIPI questionnaire at the end of follow up is expected to reflect patients’ enduring personality characteristics. Nonetheless, accumulating evidence suggests that personality traits can be treatment responsive (e.g. through the unified protocol cognitive behavioral therapy for emotional instability), which presents further opportunities to optimize weight loss outcomes [10]. Given its rapid administration via the Ten-Item Personality Inventory (TIPI), emotional stability assessment can be seamlessly integrated into pre-procedural evaluations to support personalized, risk-stratified care. Patients with lower emotional stability scores may benefit from enhanced behavioral support, including psychological counseling, closer post-procedural follow-up, or pharmacological augmentation.

Limitations of our study include modest sample sizes, which limits the precision of regression estimates and introduces possibility of false negative findings for the other personality domains, predominantly female composition, and potential selection bias from optional survey completion. Prospective studies are needed to clarify mechanisms linking emotional stability to weight outcomes. Despite these limitations, this study provides the first validated evidence that emotional stability, measurable via a brief survey, predicts ESG weight loss across diverse populations. Incorporating personality assessment into routine evaluation may enable early identification of patients who could benefit from personalized psychological support, improving long-term outcomes.

## Data Availability

All data supporting the findings of this study are available within the paper and its Supplementary Information.
